# Sequential STING and CD40 agonism drives massive expansion of tumor-specific T cells in liposomal peptide vaccines

**DOI:** 10.1038/s41423-024-01249-4

**Published:** 2025-01-01

**Authors:** Dmitrij Ostroumov, Naomi Benne, Fernando Lozano Vigario, Oscar Escalona-Rayo, Ksenia Dodz, Sarah Sauer, Lena Luisa Suhl, Hans Heiner Wedemeyer, Florian Kühnel, Bram Slütter, Thomas Christian Wirth

**Affiliations:** 1 Department of Gastroenterology, Hepatology, Infectious Diseases and Endocrinology, Hannover Medical School, Hannover, Germany; 2 Department of Infectious Diseases and Immunology, Faculty of Veterinary Medicine, Utrecht University, Utrecht, The Netherlands; 3 Division of BioTherapeutics, Leiden Academic Centre for Drug Research, Leiden, The Netherlands; 4 Department of Supramolecular and Biomaterials Chemistry, Leiden Institute of Chemistry, Leiden University, Leiden, The Netherlands

**Keywords:** Cancer, Liposomes, Heterologous vaccines, STING agonist, Costimulatory antibodies, Tumour vaccines, Gastrointestinal cancer

## Abstract

The clinical use of cancer vaccines is hampered by the low magnitude of induced T-cell responses and the need for repetitive antigen stimulation. Here, we demonstrate that liposomal formulations with incorporated STING agonists are optimally suited to deliver peptide antigens to dendritic cells in vivo and to activate dendritic cells in secondary lymphoid organs. One week after liposomal priming, systemic administration of peptides and a costimulatory agonistic CD40 antibody enables ultrarapid expansion of T cells, resulting in massive expansion of tumor-specific T cells in the peripheral blood two weeks after priming. In the MC-38 colon cancer model, this synthetic prime-boost regimen induces rapid regression and cure of large established subcutaneous cancers via the use of a single tumor-specific neoantigen. These experiments demonstrate the feasibility of liposome-based heterologous vaccination regimens to increase the therapeutic efficacy of peptide vaccines in the context of immunogenic adjuvants and costimulatory booster immunizations. Our results provide a rationale for the further development of modern liposomal peptide vaccines for cancer therapy.

## Introduction

Since the publication of the first successful phase III trial in melanoma patients in 2010 [1], immunotherapy has had a fundamental impact on cancer patients worldwide. In intrinsically immunogenic cancer entities, including melanoma and lung cancer, checkpoint inhibition has resulted in long-term remissions and even cures in patients with advanced disease [2, 3]. The clinical success of checkpoint inhibitors highlights the importance of the immunologic synapse, the location of interaction between dendritic cells and both CD4+ and CD8+ T cells, for the induction and maintenance of antitumoral immune responses [4]. In this synapse, CD8+ T cells and dendritic cells interact via a number of different molecules with T-cell-activating (costimulatory) or T-cell-inhibiting (coinhibitory) functions, the balance of which determines the final activation state of the tumor-reactive T cells [5]. Among these two receptor families with opposing functional features, only coinhibitory molecules (e.g., antagonistic antibodies targeting PD-1 and CTLA-4) have met the expectations of therapeutic targets in clinical trials. In contrast, clinical trials with agonistic antibodies directed against costimulatory molecules have failed in early clinical trials, despite their ability to activate CD8+ T cells in preclinical cancer models [6]. In theory, agonistic costimulatory antibodies have multiple potential clinical applications. Their T-cell-activating properties not only support their use as unspecific stimulators of the adaptive immune system—similar to the use of antagonistic antibodies targeting coinhibitory molecules—but also as adjuvants for vaccines that aim at inducing de novo T-cell responses [7].

With a few exceptions, current vaccines do not make use of the T-cell stimulating properties of costimulatory antibodies. Instead, most vaccine formulations employing DNA, peptides or proteins rely on the help of toll-like receptor (TLR) agonists, which typically induce weak immune responses and require multiple immunizations [8, 9]. The success of mRNA/LNP compounds as SARS-CoV-2 vaccines has heralded a new era in vaccine development due to the robust induction of humoral and cellular immune responses [10]. A major advantage of these novel coronavirus vaccines is the combination of LNPs as antigen carriers, whose physicochemical properties can be individually tailored to target antigen-presenting cells with immunogenic mRNAs that activate the targeted dendritic cells. However, even with LNP/mRNA vaccines, most study protocols employ multiple immunizations [11], which increases the risk for replicative senescence and terminal T-cell differentiation [12].

Recently, two vaccines employing peptides and LNPs/mRNAs, respectively, have received breakthrough therapy designation by the FDA for exceptional therapeutic efficacy in cancer patients (see [13] and the unpublished KEYNOTE-942 study). In the MM1636 trial, peptides targeting IDO and PD-L1 significantly increased the therapeutic efficacy of checkpoint inhibition despite the low magnitude of the induced tumor-specific T-cell response.

Here, we sought to develop a universal vaccination regimen that enables high-magnitude, ultrarapid T-cell expansion of liposomal peptide vaccines. We tested the ability of liposomal formulations (LSs) with incorporated adjuvants to increase the immunogenicity of peptide vaccines. To achieve ultrarapid T-cell amplification with only two immunizations, we combined liposomal priming with an agonistic CD40-specific antibody and tested these heterologous vaccines in a murine model of colon cancer. Our results demonstrate the feasibility of using liposomes as peptide carriers, confirm the exceptional ability of agonistic antibodies to increase T-cell responses, and support the use of heterologous vaccination regimens in future vaccine trials.

## Results

### Heterologous prime-boost vaccination against the cancer neoepitope

The goal of this research project was the development of a vaccination regimen that consists of synthetic components and allows for rapid and effective immunotherapeutic treatment of cancer patients. Previously, we demonstrated that a heterologous vaccine based on priming with peptide-pulsed dendritic cells (DCs), followed by boosting with a costimulatory agonistic CD40 antibody (Co), synthetic peptide antigen (A) and the TLR3 agonist PolyI:C (T), hereafter referred to as DC-CoAT, was able to generate massive antigen-specific CD8 T-cell responses against cancer neoepitopes within 2 weeks [7].

To develop a fully synthetic prime-boost regimen with high immunogenic potential and to alleviate the need to isolate patient-specific antigen-presenting cells, we aimed to replace the DC-priming regimen with synthetic LSs. Compared with other synthetic compounds, liposomes have been shown to be ideally suited for incorporating peptides and effectively delivering antigen cargo to antigen-presenting cells. On the basis of previous results, we employed DSPC:DPTAP:cholesterol LSs for the encapsulation of synthetic peptides to generate a specific T-cell response [14].

**Fig. 1:**
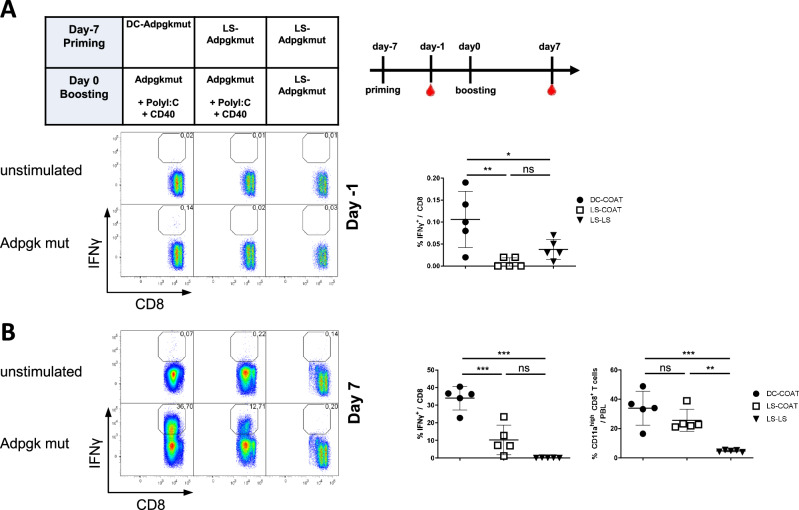
Heterologous liposomal vaccination induces a neoepitope-specific CD8 T-cell response. **A** Different primary immunizations were applied intravenously to naive C57BL/6J mice on day -7, followed by a booster immunization 1 week later. Left panel: Frequency of specific CD8+ T cells in the peripheral blood on day -1. Specific T cells were detected by intracellular IFNγ staining after restimulation with the neoantigen peptide Adpgk^mut^. Dot plots show representative examples from each group, and the numbers indicate the frequency of specific CD8+ T cells among the total CD8+ T cells. Right panel: Scatter plot of the results for IFN gamma producing specific CD8+ T cells in the peripheral blood of all the mice. **B** Left panel: Peripheral blood samples collected on day 7 after the boost were analyzed by flow cytometry and intracellular IFNγ staining. Dot plots show representative examples from each group. Right panel: Scatter plot of the results from all mice for IFN gamma producing specific CD8 T cells and total activated CD8 T cells (CD11a^hi^ CD8^int^), respectively. The bars indicate the means ± SDs of 5 samples per group. *p* ≤ 0.05 = *, *p* ≤ 0.01 = **, *p* ≤ 0.001 = ***, *p* ≤ 0.0001 = **** (one-way ANOVA with Tukey’s multiple comparison post-hoc test)

To compare the immunogenic properties of dendritic cells directly with those of LSs, C57BL/6J mice received either primary DC or LS immunizations, followed by CoAT booster vaccinations (DC-CoAT and LS-CoAT, respectively) (Fig. 1A). We compared different prime-boost regimens and analyzed the induced cancer-specific CD8 T-cell response against the well-established Adpgk^mut^ MHC class I epitope, the naturally occurring dominant neoantigen derived from the murine colon cancer cell line MC-38 [15]. To compare these heterologous, costimulation-driven vaccines with conventional homologous liposomal peptide vaccines, we included an experimental group with LS priming and LS boosting (LS-LS). We analyzed the induced T-cell response in the peripheral blood of immunized mice 1 day before the boost (Fig. 1A) and 7 days after the boost (Fig. 1B) via flow cytometry. As shown previously, DC-CoAT induced low but detectable primary immune responses that exceeded those induced by the liposomal vaccines. Following booster immunization, DC-CoAT vaccination resulted in very high antigen-specific CD8+ T-cell responses of up to 40% Adpgk^mut^-specific CD8+ T cells during the secondary effector phase (Fig. 1B). The strength of the specific CD8 T-cell response in the LS-CoAT group approximated one-third of the response in the DC-CoAT group and could be readily detected in peripheral blood, whereas the LS-LS-treated animals presented a T-cell response close to the limit of detection. Analysis of total antigen-experienced CD11a^hi^ CD8^int^ T cells [16] revealed similar frequencies of activated CD8 T cells in DC-CoAT- and LS-CoAT-treated animals but only low frequencies in mice that had received LS-LS. These results indicate that, compared with homologous liposomal vaccines, prime-boost vaccinations with costimulatory booster immunizations induce superior immune responses. Liposomal primary immunizations are able to replace dendritic cells as primary immunizations but do not reach the same magnitude when they are administered without adjuvants.

### STING agonists improve liposome-mediated dendritic cell activation and amplify heterologous LS-CoAT vaccinations

**Fig. 2:**
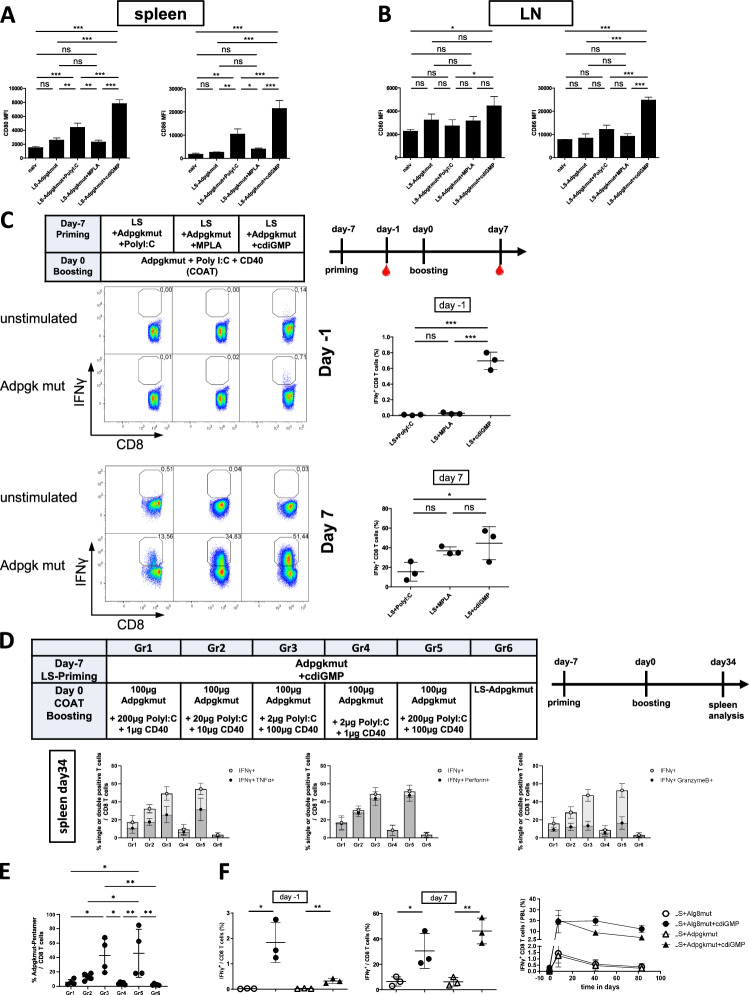
Liposomal formulations with cdiGMP enhance dendritic cell activation and induce potent neoepitop-specific CD8 T-cell responses. **A** C57BL/6J mice were immunized on day -7 with different liposomal formulations containing different adjuvants. Mice immunized with liposomes without adjuvants and naive mice are shown as reference controls. One day after immunization, dendritic cell activation in the spleen and lymph nodes was analyzed. The bar graphs show the mean MFI values of CD80 and CD86 expression on the surface of the dendritic cells. **B** Analysis of the CD80 and CD86 MFI values in the lymph nodes from the same mice described in (**A**). **C** C57BL/6J mice received primary immunization with different liposomal formulations followed by CoAT-boosting immunizations, which consisted of soluble neoantigen peptide (Adpgk^mut^), PolyI:C, and agonistic CD40 antibody, one week later. The timeline indicates measurements of samples from immunized animals. Dot plots show representative mice from the respective groups, and the numbers represent the frequency of specific CD8+ T cells among the total CD8+ T cells. Scatter plots showing the results from all the mice for the specific CD8+ T-cell response. The upper panel shows the primary immune response on day -1, and the lower panel shows the secondary immune response on day 7. The bars indicate the means ± SDs of 3 samples per group. *p* ≤ 0.05 = *, *p* ≤ 0.01 = **, *p* ≤ 0.001 = ***, *p* ≤ 0.0001 = **** (one-way ANOVA with Tukey’s multiple comparison post-hoc test). **D** C57BL/6J mice received primary immunization with liposomes containing the Adpgk^mut^ peptide and cdiGMP, followed by CoAT-boosting immunizations with the Adpgk^mut^ peptide and different CD40 antibodies and PolyI:C amounts or liposomes with the Adpgk^mut^ peptide and cdiGMP, respectively. The timeline indicates measurements of samples from immunized animals. On day 34, the polyfunctionality of Adpgk^mut^-specific CD8 T cells in the spleen was investigated via intracellular staining for IFNγ, TNFα, perforin, and granzyme B (Supplemental Fig. 2A‒C). The overlaid bar graphs show the frequency of Adpgk^mut^-specific CD8 T cells (single-positive for IFNγ or double-positive for IFNγ and TNFα, IFNγ and perforin, or IFNγ and granzyme B). The bars indicate the means ± SDs of 5 samples per group. **E** Adpgk^mut^-pentamer staining was performed on day 34 using cells from the spleens of the same animals described in (**D**). **F** Four groups of mice received the LS-CoAT prime-boost regimen with different combinations of antigen and adjuvants. Scatter plots showing antigen-specific CD8 T-cell responses on day -1 and day 7. The bars indicate the means ± SDs of 3 samples per group. *p* ≤ 0.05 = *, *p* ≤ 0.01 = **, *p* ≤ 0.001 = ***, *p* ≤ 0.0001 = **** (two-tailed t test). Right graph: Longitudinal analysis of CD8 T-cell kinetics in peripheral blood from the same experiment

We previously showed that the quantity and quality of the primary immune response are critical in prime-boost vaccination with costimulatory boosts[7]. To increase the strength of the T-cell response after LS-CoAT immunization, we intended to improve the primary T-cell response generated by LS priming. Since the activated dendritic cells used for primary immunization in the DC-CoAT vaccine induced superior T-cell expansion compared with that in the LS-CoAT vaccine, we reasoned that increased activation of DCs by the LSs might allow for a stronger primary and secondary T-cell response. For this purpose, we conducted in vitro stimulation of bone marrow-derived dendritic cells (BMDCs) with various concentrations of different adjuvants and compared the effects of the TLR3 agonist Poly I:C, the TLR4 agonist MPLA, and the stimulator of interferon genes (STING) agonist c-di-GMP (Supplemental Fig. 1A). All adjuvants and concentrations resulted in increased activation of BMDCs, as measured by CD80 and MHCII upregulation. The STING agonist resulted in the highest upregulation of these activation markers. Next, we compared the potential of different adjuvants encapsulated inside the LSs with respect to their ability to activate dendritic cells in vivo. Owing to their immunogenic and DC-activating features as well as their favorable physicochemical properties for incorporation into liposomes, we chose the TLR3 agonist Poly I:C, the TLR4 agonist MPLA, and the STING agonist c-di-GMP. LSs containing the Adpgk^mut^ antigen and adjuvants were injected into mice intravenously, and one day after LS priming, the dendritic cells from the spleen and lymph nodes were harvested and analyzed by flow cytometry for markers of DC activation. We detected significant upregulation of the costimulatory molecules CD80 and CD86 on the surface of DCs in the spleens of animals that received LS/Poly I:C or LS/c-di-GMP compared with those in the spleens of mice that received LS without adjuvants or in those of naive mice (Fig. 2A). In contrast, the increase in CD80/CD86 expression in the LS/MPLA group was not significant. Analysis of dendritic cells in the lymph nodes of the same animals revealed significant upregulation of CD80/CD86 in only the LS/c-di-GMP group (Fig. 2B). These results suggest that agonists encapsulated in liposomes enhance dendritic cell activation in secondary lymphoid organs, with the most robust activation induced by Poly I:C and c-di-GMP.

To test whether increased dendritic cell activation translates into enhanced T-cell responses, we used the same liposome/agonist combinations in heterologous, costimulatory prime-boost vaccines. Following primary liposomal immunization with either Poly I:C, MPLA, or c-di-GMP as an adjuvant, only the use of c-di-GMP resulted in detectable primary effector T cells in the peripheral blood (Fig. 2C, upper panel). After CoAT boosting, high frequencies of antigen-specific CD8+ T cells were detected in all groups, with the highest frequency detected in the LS/c-di-GMP group (Fig. 2C), which reached levels of antigen-specific CD8+ T cells similar to those observed after DC priming (Fig. 1B).

To elucidate how PolyI:C or CD40 differentially impacts T-cell expansion, titrations of PolyI:C and CD40 were performed while the amount of peptide was kept constant (Fig. 2D, upper box). A homologous LS-Adpgkmut+cdiGMP vaccination was used as a control. As shown in Fig. 2D and in the detailed T-cell kinetics (Supplemental Fig 2), both the anti-CD40 antibody and Poly I:C contributed to enhanced immune responses, but the impact of the costimulatory antibody was superior to the effects of the TLR agonist. The polyfunctionality of specific CD8+ T cells was investigated in the spleen 34 days after the boost, revealing that most of the specific CD8+ T cells were double positive for IFNγ and perforin, with half of them being double positive for IFNγ and TNFα and approximately one third being double positive for IFNγ and Granzyme B. We additionally performed pentamer staining for Adpgk^mut^-specific CD8+ T cells and detected comparable frequencies and absolute numbers of Adpgk^mut^-pentamer-positive CD8+ T cells (Fig. 2E, Supplemental Fig. 2D, E). Furthermore, we addressed potential hepatotoxicity by measuring liver transaminases on days 2 and 14 (Supplemental Fig. 2F). In all the groups, transaminase levels were not elevated and were similar to those in the naive controls.

To monitor immune responses longitudinally from initial priming until memory formation, we again performed LS CoAT and LS/c-diGMP CoAT immunizations for Adpgk^mut^ and another cancer neoepitope (Alg8^mut^) and found that c-di-GMP-encapsulated liposomes favored not only the generation of higher frequencies of primary CD8 T cells but also higher frequencies of secondary T cells after CoAT boosting (Fig. 2F). Longitudinal measurements from peripheral blood revealed long-lasting T-cell responses and slower contraction in the LS/c-di-GMP CoAT group than in the liposomal vaccination without c-di-GMP group. These results indicate that adding c-di-GMP as an adjuvant to liposomal antigen formulations is ideally suited to increase the magnitude of T-cell responses in heterologous, costimulatory vaccines and to generate robust and stable memory T cells.

### LS-COAT vaccinations enable rapid simultaneous induction of immune responses against MHC class I and II epitopes

Following the successful induction of CD8 T-cell responses via LS/c-di-GMP-CoAT, we next tested the feasibility of using LS-CoAT to induce immune responses with MHC class I epitopes of different lengths and additional MHC class II epitopes. Owing to conflicting data, it is currently unknown whether short, processed peptides (9–11 amino acids) or longer peptides, which require intracellular processing by the proteasome and the endoplasmic reticulum, are best suited for cancer vaccines [17, 18]. MHC class II epitopes have recently been shown to play a major role in antitumoral immune responses in cancer patients, both by enhancing CD8+ T-cell responses and by inducing tumor-targeting CD4+ T cells [19]. To identify the optimal composition of LS/c-di-GMP-CoAT vaccinations, we tested several combinations of long or short MHC class I epitopes with or without additional administration of liposomes containing the MHC class II epitope MTAG85B from *Mycobacterium tuberculosis*. In the last group, a fusion peptide of Adpgk^mut^ and MTAG85B was generated and incorporated into liposomes to test whether the presence of both epitopes on a single polypeptide influenced the magnitude of the immune response.

**Fig. 3:**
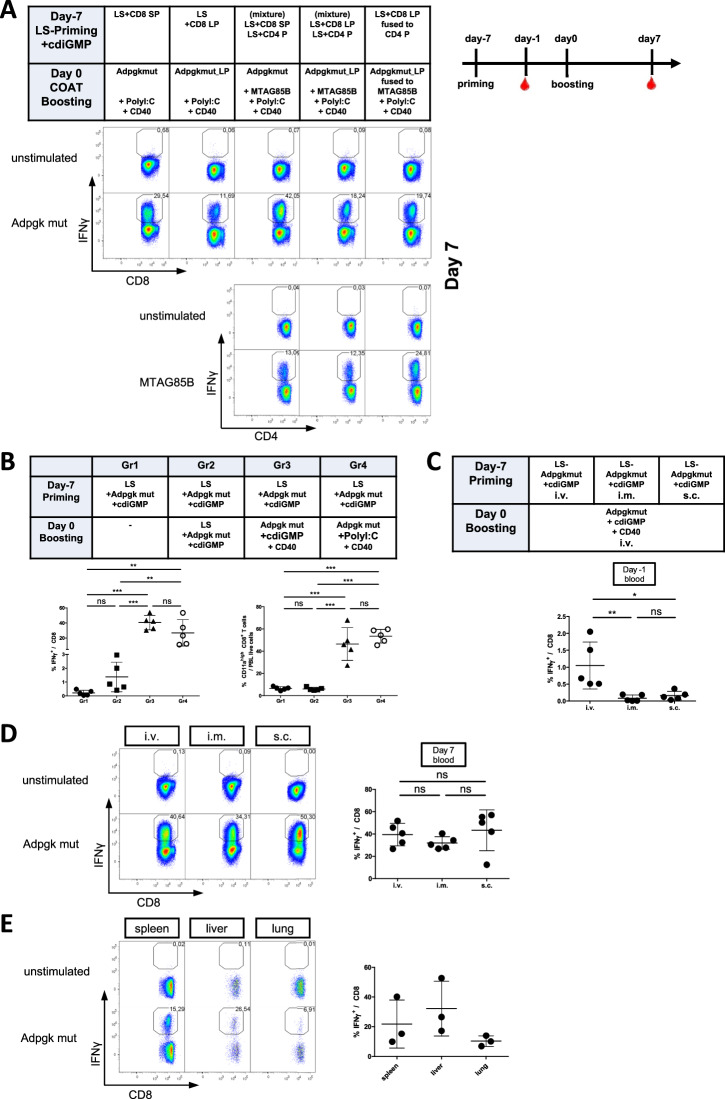
LS-CoAT vaccination generates simultaneous specific CD4+ and CD8+ T-cell responses. **A** C57BL/6J mice were immunized with liposomal formulations containing different peptides: short CD8 peptide (CD8 SP) or long 23 amino acid CD8 peptide (CD8 LP) ± liposomes containing an additional CD4 MTAG85B peptide (CD4 P). One group of mice was immunized with a 39 amino acid polypeptide that contained the Adpgk^mut^ long peptide fused to the CD4 peptide MTAG85B (CD8 LP fused to CD4 P). Representative dot plots showing specific T-cell responses in peripheral blood on day 7 (measured by ICS for IFNγ from peripheral blood). The timeline indicates measurements of samples from immunized animals. **B** Mice received the indicated immunization regimens on days -7 and 0. Scatter plot showing the specific CD8+ T-cell response in total CD8+ T cells and total activated CD8+ T cells (CD11a^hi^ CD8^int^) on day 7. **C** Mice received primary liposomal immunizations on day -7 by intravenous, intramuscular or subcutaneous injection. All the groups received intravenous CoAT boosting 1 week later. Scatter plots showing the results from all the mice for the specific CD8+ T-cell response on day -1. The bars indicate the means ± SDs of 5 samples per group. *p* ≤ 0.05 = *, *p* ≤ 0.01 = **, *p* ≤ 0.001 = ***, *p* ≤ 0.0001 = **** (one-way ANOVA with Tukey’s multiple comparison post-hoc test). **D** Representative dot plots and scatter plots from the same experiment as in (**C**) showing the immune response of the mice on day 7. **E** Organ distribution of antigen-specific CD8+ T cells from mice in the same experiment on day 76 after heterologous vaccination. Representative dot plots showing specific CD8 T-cell responses in different tissues measured by the ICS for IFNγ. Scatter plot showing the results from all the mice for the specific CD8+ T-cell response in different tissues. The bars indicate the means ± SDs of 3 samples per group

Similar to what we previously reported for DC-CoAT vaccinations, LS/c-di-GMP-CoAT vaccinations generated stronger CD8 T-cell responses after immunization with short 9mer Adpgk^mut^ peptides (SPs) than after immunization with the longer 23mer polypeptide (LP; see Fig. 3A). When liposomes with the encapsulated 16mer MHC class II epitope MTAG85 were added to liposomes containing either the short or the long Adpgk^mut^ peptide, these mixed MHC class I/II liposomes led to increased frequencies of specific CD8 T cells in both the SP and the LP groups. This enhanced CD8 T-cell response was not observed when MTAG85 was fused to Adpgk^mut^ in a single polypeptide. However, the MTAG85B-specific CD4 T-cell response was higher in the mice that received immunization with the fusion polypeptide than in the groups in which the two epitopes were administered in separate liposomes. This result suggests that coadministration of MHC class II epitopes enhances CD8 T-cell responses but that MHC class I epitopes enhance CD4 T-cell responses only when MHC class I and II epitopes are present on the same polypeptide.

We next sought to determine how the use of c-di-GMP as an adjuvant for booster immunization influences the magnitude of the CD8+ T-cell response. Although there was a trend toward a higher frequency of CD8 T cells after homologous LS/c-di-GMP vaccination, it was not significantly higher compared to a single LS/c-di-GMP immunization (Fig. 3B), indicating that c-di-GMP without costimulation is not sufficient for robust secondary CD8 T-cell expansion. However, we still reasoned that c-di-GMP could substitute for Poly I:C when agonistic costimulatory antibodies are coadministered. Indeed, CoAST-boosting (**Co**stimulation, **a**ntigen, and **ST**ING agonist) reached frequencies of specific CD8 T cells comparable to those of the CoAT booster with Poly I:C and showed similar frequencies of activated CD8 T cells (based on the CD11a^hi^ CD8^int^). We then tested different routes of application (i.v., i.m., s.c.) for the LS-CoAST vaccination regimen (Fig. 3C). While significantly more specific CD8+ T cells could be detected in the blood of intravenously immunized mice in the primary effector phase, the subsequent i.v. CoAST boosting in all groups equalized the frequencies of specific CD8+ T cells in the peripheral blood for all groups (Fig. 3D). To analyze the trafficking pattern of the induced CD8+ T-cell response, we compared the distribution of the generated T-cell response in different organs and observed similar high frequencies of specific CD8+ T cells in the spleen, liver and lung at a memory time point (day 76 after the boost) (Fig. 3E). In summary, heterologous, liposomal CD8 T-cell vaccines can be enhanced with the help of MHC class II epitopes. While c-di-GMP enhances the primary CD8+ T-cell response, it is not superior to Poly I:C in booster vaccination and is unable to replace the agonistic anti-CD40 antibody.

### LS-CoAT vaccination induces therapeutic CD8+ T-cell responses in murine cancer models

**Fig. 4:**
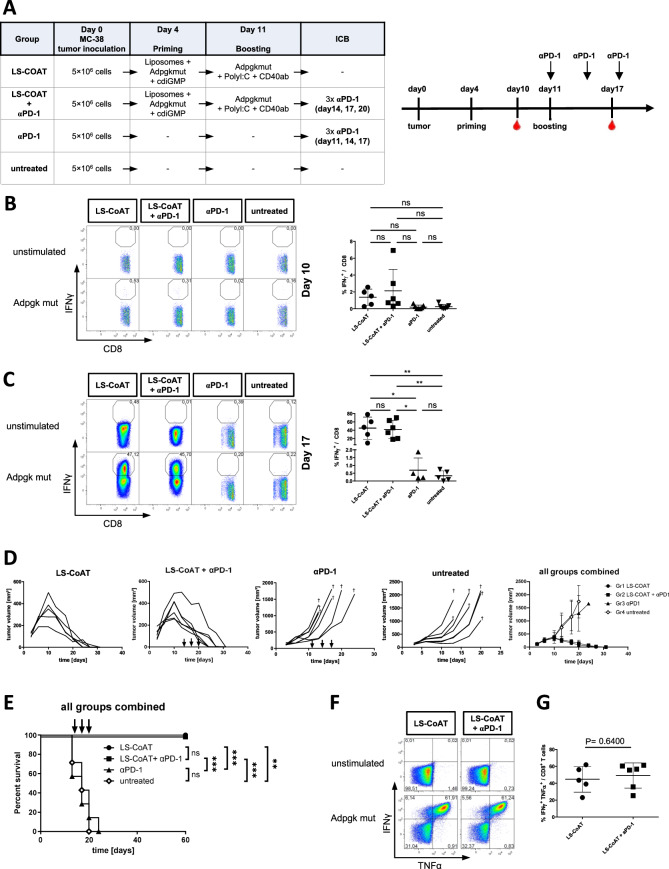
Therapeutic LS-CoAT vaccination induces complete regression of established tumors. **A** Experimental setup. C57BL/6J mice were inoculated subcutaneously with MC-38 cells on day 0. Tumor-bearing mice received either LS-CoAT vaccination, anti-PD-1 antibody, or LS-CoAT vaccination plus anti-PD-1 antibody. Untreated tumor-bearing mice were used as controls. The timeline indicates measurements of samples from immunized animals. The vertical arrows indicate αPD-1 applications. **B** Left panel: Representative dot plots showing the specific CD8+ T-cell response in peripheral blood on day 10 (measured by ICS for IFNγ). Right panel: Scatter plot showing the antigen-specific T-cell response of all the mice; statistical significance for all the groups was calculated by ANOVA = * (*P* = 0,0372), followed by Tukey’s multiple comparison post-hoc test. The bars indicate the means ± SDs of 5–7 samples per group. *p* ≤ 0.05 = *, *p* ≤ 0.01 = **, *p* ≤ 0.001 = ***, *p* ≤ 0.0001 = ****. **C** Immune response from the same experiment as in (**B**) on day 17. **D** Individual, longitudinal tumor growth measurements in mice from all groups and a combined graph with mean tumor volumes from all groups are shown. The arrows indicate the application of the anti-PD-1 antibody in the different groups. **E** Combined survival curves of mice from the same experiment. The arrows indicate the application of the anti-PD-1 antibody in the different groups. For comparison of survival curves, the log-rank (Mantel‒Cox) test was applied. **F** Polyfunctionality of the induced Adpgk^mut^-specific CD8+ T-cell response was analyzed via intracellular staining for IFNγ and TNFα (using ICS; gated on live CD8+ T cells). Representative dot plots showing specific T-cell responses in peripheral blood from the respective groups on day 56 after tumor inoculation. **G** Scatter plot showing specific CD8 T-cell responses (IFNγ+ and TNFα+) in total CD8 T cells from all the mice shown in (**F**). The bars indicate the means ± SDs of 3 samples per group. *p* ≤ 0.05 = *, *p* ≤ 0.01 = **, *p* ≤ 0.001 = ***, *p* ≤ 0.0001 = **** (two-tailed *t* test)

To evaluate the therapeutic efficacy of the LS-CoAT vaccination regimen against cancer, we used a well-established MC-38 colon cancer model of subcutaneous tumors (Fig. 4A). In this study, we investigated whether immune checkpoint blockade of PD-1 could provide additional survival benefits for mice treated with LS-CoAT. Anti-PD-1 monotherapy and untreated control mice were included as additional comparison groups in this experiment. MC-38 tumor-bearing mice were vaccinated with LS-CoAT targeting Adpgk^mut^, the naturally occurring dominant neoantigen in MC-38 cancer cells [15]. On day 10 after tumor implantation, there were no detectable immune responses in the untreated control group or the group receiving checkpoint inhibition, whereas low frequencies of Adpgk^mut^-specific CD8+ T cells were detected in the groups that received LS priming (Fig. 4B). After the CoAT boost, the induced T-cell response was relatively high in the LS-CoAT and LS-CoAT +αPD-1 groups, whereas the αPD-1 monotherapy group and untreated animals continued to have only low frequencies of tumor-specific CD8+ T cells in the peripheral blood (Fig. 4C). Tumor growth kinetics were very similar in the LS-CoAT and LS-CoAT+αPD-1 groups, with complete regression of all tumors after the boost (Fig. 4D). The subcutaneous tumor growth of the αPD-1-treated and untreated control mice did not differ, and the mice were euthanized because of continuous tumor growth. Survival analysis revealed significant differences between the LS-CoAT and LS-CoAT+αPD-1 groups and the αPD-1 and untreated control groups (Fig. 4E). Compared with no PD-1 blockade, monotherapy with anti-PD-1 blockade did not prolong the survival of mice. Functional analysis of the induced T-cell response in the blood of LS-CoAT-vaccinated animals revealed comparable frequencies of IFNγ/TNFα double-positive Adpgk^mut^-specific CD8 T cells after complete tumor regression (Fig. 4F, G), indicating fully functional cytotoxic effector T cells.

We additionally evaluated LS-CoAT therapeutic vaccination in a second tumor model in which we implanted the murine hepatoma cell line Hep-55.1C expressing the Adpgk^mut^ epitope. Animals were treated either with LS-CoAT and anti-PD-1 antibody or with anti-PD-1 antibody. Untreated tumor-bearing mice were included as an additional control group (Supplemental Fig. 3A). In this model, we analyzed T-cell trafficking via Adpgk^mut^ pentamer staining to investigate the tumor-specific CD8+ T-cell response in draining lymph nodes and tumors at the time-point of euthanization (Supplemental Fig. 3B). While there were no detectable differences in the tumor-draining lymph nodes, the intratumoral frequencies of specific T cells in the combination LS-CoAT + αPD-1 group were almost twofold higher than those in the αPD-1 monotherapy group and almost 8-fold higher than those in the untreated animals. PD-1 expression in tumors was higher than that in lymph nodes but was similar in all groups (Supplemental Fig. 3C). Analysis of T-cell polyfunctionality (detected by ICS for the effector molecules IFNγ, TNFα, perforin, and granzyme B) and the degranulation marker CD107a in the spleen, draining lymph node and tumor revealed signs of T-cell exhaustion in the group that received only αPD-1 therapy and in the untreated group (Supplemental Fig. 3D–F). Overall, the frequencies of Adpgk^mut^-specific T cells detected by ICS resembled those detected via pentamer staining in the LS-CoAT + PD-1 group, while the pentamer frequencies were substantially higher than the ICS frequencies in the αPD-1 monotherapy group and untreated group, indicating substantial exhaustion.

With respect to T-cell responses in peripheral blood, we detected kinetics similar to those observed for the MC-38 experiment with LS-CoAT immune responses exeeding PD-1 monotherapy and the control group. However, we observed significantly lower T-cell frequencies in the LS-CoAT + αPD-1 group compared to LS-CoAT group on day 21, indicating that the addition of PD-1 did not increase but rather decreased T-cell frequencies (Supplemental Fig. 4A–E). Adpgk^mut^-specific T cells recognized not only the Adpgk^mut^ peptide in vitro but also vital Hep-55.1C tumor cells. Similar to the MC-38 experiments, the survival of the LS-CoAT-treated mice was superior to that of the PD-1 therapy-treated and untreated control mice (Supplemental Fig. 4F). PD-1 administration negatively impacted LS-CoAT, although the difference between the two groups was not significant.

Since CD40 antibodies and Poly I:C by themselves are tumoricidal, we compared our LS-CoAT vaccination with LS-CoT, an LS-CoAT variation without Adpgk^mut^ peptide administration (Supplemental Fig. 5). LS-CoAT induced substantially higher Adpgk^mut^-specific immune responses than LS-CoT. Compared with LS-CoT treatment, therapeutic vaccination with LS-CoAT significantly prolonged the survival of mice bearing aggressively growing tumors (Supplemental Fig. 5D), suggesting the need for a tumor-specific antigen as a treatment target.

These cancer vaccines demonstrate the potential of LS-CoAT immunizations to treat large tumors with a single MHC class I target antigen. The antitumoral effects of checkpoint inhibitors are inferior to those of LS-CoAT and are unable to further enhance the therapeutic efficacy of LS-CoAT in combination therapies.

## Discussion

The advent of novel immunotherapies, including checkpoint inhibitors, CAR-T cells, and bispecific antibodies, has revolutionized the therapy of cancer patients. Despite the overwhelming success of checkpoint inhibitors, therapeutic cancer vaccines have not been able to enter daily clinical practice, primarily because of low clinical efficacy and high costs. Recent trials with peptide and LNP/mRNA vaccines ([13] and KEYNOTE-942, unpublished data) in combination with checkpoint inhibition for the treatment of melanoma patients, however, have shown remarkable clinical efficacy and led to the first Fast Track and Breakthrough Designations granted by the Federal Drug Administration (FDA) and Prime Designation granted by the European Medicines Agency. In these recent breakthrough clinical trials, both peptides and mRNAs were used as antigen carriers, and vaccines were administered multiple times to achieve the desired magnitude of the adaptive immune response.

Here, we demonstrate that heterologous peptide vaccines are able to induce massive immune responses with only two immunizations when used in LSs and heterologous vaccinations with costimulatory booster immunizations. In previous publications, we were able to show the exceptional ability of the CoAT (**co**stimulation, peptide as an **a**ntigen, **T**LR agonist) booster vaccination to amplify primary immune responses [7]. However, to date, we have failed to establish a synthetic primary vaccine that was able to replace dendritic cell immunizations in the highly effective DC-CoAT vaccines. The results of the current study demonstrate that, similar to mRNA-carrying lipid nanoparticles, liposomes are ideally suited as carrier vehicles for peptides to induce robust primary T-cell responses. In contrast to mRNA-carrying lipid nanoparticles, however, liposome/peptide combinations require an additional adjuvant for maximum immunogenicity, and we showed that STING agonists represent ideal adjuvants for incorporation into peptide-bearing liposomes. Our results corroborate findings by others that the STING pathway drives type I interferon production and contributes to the initiation of adaptive anticancer responses, particularly when it is incorporated into liposomes [20, 21]. In contrast to most synthetic compounds, liposomes can be individually tailored to either target dendritic cells directly or be taken up by them, which constitutes a prerequisite for the induction of efficacious adaptive immune responses. It is tempting to speculate that this ability to effectively deliver antigen cargo to dendritic cells makes liposomes unique among all synthetic antigen carriers and therefore ideal vehicles for a large variety of different antigen formulations.

Most notably, the use of the liposome/peptide/c-di-GMP complexes allows for booster immunization as early as 7 days later, resulting in massive secondary T-cell expansion and peak frequencies of more than 40% antigen-specific CD8+ T cells in the peripheral blood 14 days after primary immunization. According to the model originally proposed by Badovinac and Harty [22], the LS-CoAT prime-boost protocol seems to be able to generate accelerated primary memory, which subsequently expands massively following CoAT booster immunizations.

In contrast to primary immunization, liposome-peptide formulations were not superior to peptide immunizations without liposomes (as in CoAT) in secondary booster immunizations. This fact likely reflects the lower activation threshold and the higher numerical abundance of (early) primary memory T cells than their naive counterparts and confirms the ability of agonistic costimulatory antibody/peptide formulations to expand previously activated T-cell populations. With regard to the adjuvant used, our results indicate that both TLR3 and STING agonists can be used in combination with costimulatory antibodies, suggesting that the choice of adjuvant is less critical for boosting than for priming.

With respect to the design of optimal prime-boost regimens, our results suggest that the quality of the primary immune response seems to be critical for the induction of robust T-cell responses. Further support for this hypothesis comes from the fact that CD4 T-cell help in the form of class II MHC peptides was able to further improve the quality of the primary immune response but not the secondary immune response (data not shown). In the weakly immunogenic primary liposomal formulation, CD4 T-cell help is expected to increase the activation of dendritic cells and support subsequent secondary memory formation. In contrast, CD4 T cells by MHC class II epitopes are dispensable for the potent costimulatory boost, where CD4 T-cell help is directly provided via the CD40/CD40L signaling pathway. Intriguingly, CD8 T cells help in turn enhance CD4 T-cell responses but only when MHC class I and II epitopes are fused in a single peptide chain. While the exact reason for this observation remains unclear, the use of fused epitopes should be considered when the magnitude of the induced CD4 T-cell response is of special interest in vaccinations.

In summary, our results corroborate the fact that agonistic costimulatory antibodies, although inefficient as monotherapies in cancer patients, represent the best means to quickly and massively amplify primary T-cell responses. This conclusion is supported by recent data showing that the inclusion of CD40L mRNA in homologous mRNA vaccines enhances CD8+ T-cell expansion [23]. As a downside to this homologous approach, repeated CD40/CD40L stimulation increases the risk for systemic side effects, including hepatitis [24]. With the use of LSs to induce primary immune responses, our heterologous LS-CoAT approach requires a single administration of an agonistic antibody to induce massive CD8+ T-cell responses, which limits the risk for potential side effects. This extraordinarily rapid and efficient induction of tumor-specific T cells with peptides instead of with mRNA formulations suggests that the immunogenicity of peptides can be vastly enhanced with the use of adjuvants and costimulatory agonists. Our combination of liposomal priming with a STING agonist and a heterologous, costimulatory boost, therefore, seems to be an important step toward the development of powerful synthetic peptide vaccines for the treatment of cancer patients.

## Materials and methods

### Animals

C57BL/6J mice (6‒8 weeks) were purchased from Charles River or bred at the Animal Care facility of the Medical School Hannover, Germany. All animal experiments were performed according to the German legal guidelines for animal care and experimentation (TierSchG) and were approved by the institutional and governmental boards (LAVES).

### Cell lines

MC-38 cells were kindly provided by Michael Neumaier (University of Mannheim, Germany). The murine hepatoma cell line Hep-55.1C (Cell Lines Service) was cultured at 37 °C and 5% CO2 in Dulbecco’s modified Eagle’s medium (DMEM) + GlutaMAX‐I (Gibco) supplemented with 10% fetal calf serum (Gibco), 100 U/mL penicillin, and 100 mg/mL streptomycin (Bio&SELL). The cells were tested for mycoplasma contamination on a regular basis.

### Retroviral transduction of murine cell lines

For the generation of retroviral particles, the following DNA plasmids were used: pQC-Adpgkmut-IP, carrying the Adpgk^mut^ epitope (original plasmid pQCXIP from Clontech (Takara Bio), pVSV-G, (containing DNA for *env*) and pMΔL (containing DNA for *gag* + *pol*)). PEI was used as a transfection agent for the virus production cell line Phoenix-Amphos (ATCC, USA). The Hep-55.1C liver cancer cell line was transduced via a standard protocol with polybren (4 μg/ml) as the transduction agent. After transduction, the cells were selected with puromycin (Carl Roth, Germany) to ensure that only Hep-55.1C cells carrying the Adpgk^mut^ epitope remained.

### Peptides

All peptides (GenScript Biotech Rijswijk, Netherlands) were dissolved in DMSO, aliquoted, and stored at −80 °C. The sequences of the peptides used are listed in Supplemental Table 1.

### Dendritic cell (DC), liposomal (LS) immunizations, CoAT vaccinations

Splenic DCs were isolated from donor mice 10‒14 days after subcutaneous injection of 5 × 10^6^ B16 cells expressing Flt3L via CD11c microbeads according to the manufacturer´s instructions (Miltenyi Biotec, Bergisch Gladbach, Germany) as described previously [25]. After the isolation of DCs, the cells were matured in vitro with LPS (0.5 μg/ml) and incubated in the presence of peptides (2 μg/ml) for approximately 2 h. For vaccination, 1 × 10^6^ DCs were injected intravenously into individual mice. Liposomes with encapsulated peptides were characterized prior to immunization. For LS immunization, liposomes containing 10 nmol of encapsulated peptides were applied intravenously. In some experiments, different application routes (i.v., i.m., s.c.) were used for LS immunization. In some groups, liposomes were coencapsulated with peptides and different adjuvants (MPLA, c-di-GMP, or Poly I:C; all from InvivoGen, San Diego, CA, USA). The amounts of liposomes coencapsulated with adjuvants that the mice received were as follows: 4.66 µg PolyI:C, 17.3 µg MPLA, and 1.97 µg c-di-GMP. For CoAT immunization, the mice were injected intravenously with a combination of 100 μg of soluble peptides (GenScript Biotech Rijswijk, Netherlands), 200 μg of Poly I:C (InvivoGen, San Diego, CA, USA) and 100 μg of agonistic anti-CD40 antibody (clone 1C10, hybridoma kindly provided by Frances Lund, Department of Microbiology, University of Alabama at Birmingham, AL, USA). In some groups, 5 µg of c-di-GMP (InvivoGen, San Diego, CA, USA) was used instead of PolyI:C for CoAST immunizations.

### Antibodies

The following antibodies were used for FACS analysis: CD8 (53–6.7), CD4 (GK1.5), CD11c (N418), interferon gamma (XMG1.2), TNFalpha (MP6-XT22), TruStain FcX (anti-mouse CD16/32) (93), CD80 (16-10A1), CD86 (GL-1), I-A/I-E (MHCII) (M5/114.15.2), Granzyme B (QA18A28), Perforin (S16009A) (from Biolegend, Amsterdam, The Netherlands), CD11a (M17/4), CD90.2 (53-2.1), CD107a (1D4B) (eBioscience, San Diego, CA, USA) and appropriate isotype controls.

### Phenotypic analysis of dendritic cells

After the mice were euthanized, the spleens and lymph nodes (inguinal, axillary, and brachial LN) were removed and then forced through 40 μm cell strains (Falcon, Colorado Springs, CO, USA) to obtain single-cell suspensions. Prior to staining, the cells were treated with TruStain FcX (BioLegend) to block Fc-receptors for 15 min at 4 °C and then stained with antibodies against extracellular proteins (CD11c, CD80, and CD86) for 20 min. Dendritic cells were identified by CD11c expression and analyzed for activation (based on CD80 and CD86 marker expression).

### Bone marrow-derived dendritic cells (BMDCs)

Bone marrow was isolated from the tibias and femurs of wild-type (WT) C57BL/6 mice. A single-cell suspension of bone marrow cells was obtained via strain over a 70 μm cell strainer (Greiner Bio-One B.V., Alphen aan den Rijn, NL). The cells were cultured for 10 days in complete IMDM (cIMDM) supplemented with 2 mM 3-glutamine, 8% v/v FCS, 100 U/mL penicillin/streptomycin, and 50 μM β-mercaptoethanol at 37 °C and 5% CO2 in 95-mm Petri dishes (Greiner Bio-One B.V., Alphen aan den Rijn, NL) supplemented with 20 ng/mL GM-CSF. The medium was changed every other day. For the in vitro activation assay, BMDCs were exposed to different concentrations of different adjuvants (PolyI:C (5, 10, 20, 50, or 100 µg/ml), MPLA (5, 10, 20, 50, or 100 ng/ml), or cdiGMP (50 µg/ml)) for 24 h. BMDCs were identified by CD11c expression, and activation was analyzed by the upregulation of CD80 and MHCII (values in geometric mean fluorescence intensity, gMFI) via flow cytometry.

### Quantification and phenotypic analysis of antigen-specific T cells

The magnitude of the epitope-specific CD8 T-cell response was determined by intracellular interferon gamma staining as previously described[22].

Synthetic peptides (Adpgk^mut^, MTAG85B, and Alg8^mut^) were used for restimulation (2 µg/ml) of samples from immunized mice. In some experiments for measuring tumor cell recognition, Hep-55.1C Adpgk^mut^-expressing cells were initially stimulated overnight with IFNγ (50 U/ml, recombinant, carrier-free, from Biolegend) to ensure sufficient MHC class I expression. Afterwards, the Hep-55.1C cells were washed with DMEM to remove the recombinant IFNγ that was used for tumor cell stimulation. For the analysis of the tumor-specific CD8 T-cell response, 0.2 × 10^6^ Hep-55.1C Adpgk^mut^-expressing cells were used for stimulation and were incubated together with samples from immunized animals. All samples (either stimulated with peptide or tumor cells) were incubated in the presence of GolgiPlug (containing brefeldin A, from BD Bioscience) overnight, followed by intracellular cytokine staining (ICS). For staining, a small volume (~50 μL) of blood was obtained via submandibular bleeding. Prior to staining, the cells were treated with TruStain FcX (BioLegend) to block Fc-receptors for 15 min at 4 °C and then stained with antibodies against extracellular proteins for 20 min, followed by ICS for interferon gamma, TNF alpha, perforin, and granzyme B using the BD Cytofix/Cytoperm Kit according to the manufacturer´s instructions (BD Bioscience, Heidelberg, Germany). Blood, tumor, total splenocytes, or other organ samples from sacrificed mice were subjected to staining. For the analysis of the organ-specific distribution of antigen-specific CD8 T cells, the mice were sacrificed and subjected to cardiac perfusion with Dulbecco´s PBS (Gibco, Germany) prior to harvesting the organs. Organs were forced through 40 μm Cell Strainers (Falcon, Colorado Springs, CO, USA) to obtain single-cell suspensions, followed by intracellular interferon gamma staining. The obtained flow cytometry data were analyzed using FlowJo software (Tree Star, Ashland, OR, USA).

### Pentamer staining

Adpgk^mut^-pentamer (conjugated to APC) was purchased from ProImmune (Oxford, UK) following a standard protocol. Briefly, pentamer and TruStain FcX were added to the cells and incubated for 10 min at room temperature, after which the remaining surface antibodies were added to the cells, followed by an additional 20 min of incubation on ice. After two washing steps with FACS buffer, the stained cells were analyzed by flow cytometry.

### Detection of hepatocyte damage

Serum transaminase measurements were performed according to the manufacturer’s instructions via an alanine transaminase (ALT) assay kit (ScienCell).

### Induction of subcutaneous cancers

For the establishment of subcutaneous colorectal cancer, 5 × 10^6^ MC-38 cells (kindly provided by Michael Neumaier, University of Mannheim, Germany) were resuspended in PBS and subcutaneously injected into the flanks of C57BL/6J mice. For the establishment of subcutaneous liver cancer, 10^7^ Hep-55.1C cells (Cell Lines Service) were resuspended in PBS and subcutaneously injected into the flanks of C57BL/6J mice. For the tumor experiments, the tumor volume was calculated via the ellipsoid formula (0.5 × length × (width)^2^), the tumor volume was measured three times a week, and the mice were sacrificed when the calculated tumor volume exceeded 1500 mm^3^.

### Treatment of subcutaneous tumor-bearing mice with blocking antibodies

A blocking antibody against PD-1 (RMP1-14) was purchased from BioXCell. For the MC-38 tumor model, the mice were injected i.p. 3 times (in the group that received LS-CoAT and anti-PD-1 treatment: on days 14, 17, and 20; in the group that received only anti-PD-1 treatment: on days 11, 14, and 17), 150 μg of anti-PD-1 diluted in 0.9% NaCl solution was used. For the Hep-55.1C tumor model, the mice were injected i.p. 2 times a week for 3 weeks (on days 7, 10, 14, 17, 21, and 24) with 150 μg of anti-PD-1 diluted in 0.9% NaCl solution.

### Generation of liposomal formulations

The lipids 1,2-distearoyl-sn-glycero-3-phosphocholine (DSPC) and 1,2-dipalmitoyl-3-trimethylammonium-propane (DPTAP) were purchased from Avanti Polar Lipids (Alabaster, AL, USA). Cholesterol was obtained from Sigma-Aldrich (Zwijndrecht, the Netherlands). Adjuvant bis-(3′-5′)-cyclic dimeric guanosine monophosphate (c-di-GMP) was purchased from InvivoGen (San Diego, CA, USA). Monophosphoryl lipid A (MPLA) was purchased from Avanti Polar Lipids (Alabaster, AL, USA). High-molecular-weight polyinosine-polycytidylic acid (HMW Poly(I:C)) was obtained from InvivoGen (San Diego, CA, USA). Whatman™ Nucleopore™ track-etched polycarbonate membranes and Vivaspin centrifugal concentrators were purchased from Sigma-Aldrich (Zwijndrecht, the Netherlands).

Liposomes were prepared via the lipid film hydration method. Briefly, a total of 10 mg of DSPC, DPTAP or cholesterol dissolved in chloroform was mixed in a round-bottom flask at a molar ratio of 4:1:2 (DSPC:DPTAP:Cholesterol). For liposomes containing adjuvants, the lipids were mixed with 150 µg of bis-(3′-5′)-cyclic dimeric guanosine monophosphate (c-di-GMP), 80 nmol MPLA or 100 µg of high-molecular-weight polyinosine-polycytidylic acid (HMW Poly(I:C)). The organic solvent was removed by means of a rotary evaporator at 40 °C for at least 30 min with pressure set at 180 mbar. The dry lipid film was hydrated via rotation with 1 mL of Milli-Q water + 0.04% NH_4_OH for empty liposomes or peptides (Adpgk^mut^, MTAG85B, Alg8^mut^, Adpgk^mut^ long, and the fusion peptide Adpgk^mut^ long/MTAG85B) dissolved in Milli-Q water + 0.04% (v/v) NH4OH for 30 min at 60 °C. Glass beads were added to the round-bottom flask during this step to facilitate hydration of the dry lipid film. The resulting suspension of multilamellar vesicles was snap-frozen in liquid N_2_ and freeze-dried overnight (Christ alpha 1-2 freeze-dryer, Osterode, Germany). The dry product was rehydrated stepwise by first adding 25% of the final volume of 10 mM phosphate buffer (PB), pH 7.4, followed by vortexing and 30 min of incubation at 60 °C in a water bath. This step was repeated once. The final rehydration step consisted of the addition of the remaining 50% of the final volume of 10 mM PB (pH 7.4), vortexing and incubation in a 60 °C water bath for at least 1 h. The lipid concentration after rehydration was 5 mg/mL. The formulation was extruded at high pressure via a LIPEX Extruder (Northern Lipids Inc., Canada) connected to a water bath at 60 °C. The formulations were extruded 4x through 400 nm and 200 nm stacked track-etched polycarbonate membranes. The resulting monodisperse formulations were purified via Vivaspin centrifugal concentrators (MWCO 100,000 Da) via centrifugation at 1500 rpm and 4 °C to remove nonencapsulated peptides and adjuvants.

### Characterization of the liposomal formulations

The z-average hydrodynamic diameter (Zave), polydispersity index (PdI), and ζ-potential of the formulations were determined via dynamic light scattering and laser Doppler electrophoresis with a Zetasizer NanoZS (Malvern Panalytical, UK).

The HMW poly(I:C) content was determined by measuring the absorbance at 260 nm via a Tecan plate reader (Salzburg, Austria).

The peptide, c-di-GMP and MPLA contents were determined via reverse-phase ultrahigh-performance liquid chromatography (UPLC). For this, 10 µL of formulation was injected into a 1.7 µm BEH C18 column (2.1 × 50 mm, Waters ACQUITY UPLC, Waters, MA, USA). The column temperature was set at 40 °C. A mobile phase linear gradient was applied to the column starting with 95% Milli-Q water with 0.1% TFA (solvent A) and 5% acetonitrile with 0.1% TFA (solvent B) and continuing to 95% solvent B for 7 min. The gradient was followed by 95% solvent B for 2 min and then returned to 95% solvent A for 3 min. The flow rate of the mobile phase was set at 0.5 mL/min. Peptides were detected by absorbance at 220 nm using an ACQUITY UPLC TUV detector. c-di-GMP was detected by absorbance at 254 nm using an ACQUITY UPLC TUV detector. MPLA was detected via an ACQUITY UPLC evaporative light scattering detector.

### Statistical analysis

Statistical significance was assessed via a two-tailed *t*-test with a confidence interval of >95% or one-way ANOVA with Tukey’s multiple comparison post-hoc test. The data are presented as the means (±SDs). For comparison of survival curves, the log-rank test was applied. The levels of significance are indicated by asterisks: *p* < 0.05 = *, *p* < 0.01 = **, *p* < 0.001 = ***, *p* < 0.0001 = ****.

## Supplementary information

Supplemental Figure 1: Different adjuvants induce the activation of cultured BMDCs

Supplemental Figure 2: Tcell expansion in heterologous vaccination is mediated mainly by CD40 costimulation

Supplemental Figure 3: Anti-PD-1 monotherapy is insufficient to prevent T-cell exhaustion in the Hep-55.1C tumor model

Supplemental Figure 4: Therapeutic vaccination with LS-CoAT induces high T-cell responses that recognize cancer cells in vitro and prolong the survival of tumor-bearing mice

Supplemental Figure 5: Tumor antigens in therapeutic T-cell vaccination are necessary for prolonged survival of tumor-bearing mice

Supplemental Figure Captions

Supplemental table 1

## References

[1] Hodi FS, O’Day SJ, McDermott DF, Weber RW, Sosman JA, Haanen JB, et al. Improved survival with ipilimumab in patients with metastatic melanoma. N Engl J Med. 2010;363:711–23. 20525992 10.1056/NEJMoa1003466 PMC3549297

[2] Farolfi A, Ridolfi L, Guidoboni M, Nicoletti SV, Piciucchi S, Valmorri L, et al. Ipilimumab in advanced melanoma: reports of long-lasting responses. Melanoma Res. 2012;22:263–70. 22516968 10.1097/CMR.0b013e328353e65c

[3] Postow MA, Callahan MK, Wolchok JD. Immune checkpoint blockade in cancer therapy. J Clin Oncol 2015;33:1974–82. 25605845 10.1200/JCO.2014.59.4358 PMC4980573

[4] Shaw AS. T-cell activation and immunologic synapse. Immunol Res. 2005;32:247–52. 16106076 10.1385/IR:32:1-3:247

[5] Chen L, Flies DB. Molecular mechanisms of T cell co-stimulation and co-inhibition. Nat Rev Immunol. 2013;13:227–42. 23470321 10.1038/nri3405 PMC3786574

[6] Baeuerle PA, Wesche H. T-cell-engaging antibodies for the treatment of solid tumors: challenges and opportunities. Curr Opin Oncol. 2022;34:552–8. 35880455 10.1097/CCO.0000000000000869 PMC9415207

[7] Nimanong S, Ostroumov D, Wingerath J, Knocke S, Woller N, Gurlevik E, et al. CD40 signaling drives potent cellular immune responses in heterologous cancer vaccinations. Cancer Res. 2017;77:1918–26. 28202532 10.1158/0008-5472.CAN-16-2089

[8] Pulendran B, Ahmed R. Immunological mechanisms of vaccination. Nat Immunol. 2011;12:509–17. 21739679 10.1038/ni.2039 PMC3253344

[9] Pulendran BS, Arunachalam P, O’Hagan DT. Emerging concepts in the science of vaccine adjuvants. Nat Rev Drug Discov. 2021;20:454–75. 33824489 10.1038/s41573-021-00163-y PMC8023785

[10] Polack FP, Thomas SJ, Kitchin N, Absalon J, Gurtman A, Lockhart S, et al. Safety and efficacy of the BNT162b2 mRNA Covid-19 vaccine. N Engl J Med. 2020;383:2603–15. 33301246 10.1056/NEJMoa2034577 PMC7745181

[11] Sahin U, Oehm P, Derhovanessian E, Jabulowsky RA, Vormehr M, Gold M, et al. An RNA vaccine drives immunity in checkpoint-inhibitor-treated melanoma. Nature. 2020;585:107–12. 32728218 10.1038/s41586-020-2537-9

[12] Wirth TC, Xue HH, Rai D, Sabel JT, Bair T, Harty JT, et al. Repetitive antigen stimulation induces stepwise transcriptome diversification but preserves a core signature of memory CD8(+) T cell differentiation. Immunity. 2010;33:128–40. 20619696 10.1016/j.immuni.2010.06.014 PMC2912220

[13] Kjeldsen JW, Lorentzen CL, Martinenaite E, Ellebaek E, Donia M, Holmstroem RB, et al. A phase 1/2 trial of an immune-modulatory vaccine against IDO/PD-L1 in combination with nivolumab in metastatic melanoma. Nat Med. 2021;27:2212–23. 34887574 10.1038/s41591-021-01544-x PMC8904254

[14] Benne N, van Duijn J, Lozano Vigario F, Leboux RJT, van Veelen P, Kuiper J, et al. Anionic 1,2-distearoyl-sn-glycero-3-phosphoglycerol (DSPG) liposomes induce antigen-specific regulatory T cells and prevent atherosclerosis in mice. J Control Release. 2018;291:135–46. 30365993 10.1016/j.jconrel.2018.10.028

[15] Yadav M, Jhunjhunwala S, Phung QT, Lupardus P, Tanguay J, Bumbaca S, et al. Predicting immunogenic tumour mutations by combining mass spectrometry and exome sequencing. Nature. 2014;515:572–6. 25428506 10.1038/nature14001

[16] Rai D, Pham NL, Harty JT, Badovinac VP. Tracking the total CD8 T cell response to infection reveals substantial discordance in magnitude and kinetics between inbred and outbred hosts. J Immunol. 2009;183:7672–81. 19933864 10.4049/jimmunol.0902874 PMC2808048

[17] Hu J, Budgeon LR, Balogh KK, Peng X, Cladel NM, Christensen ND. Long-peptide therapeutic vaccination against CRPV-induced papillomas in HLA-A2.1 transgenic rabbits. Trials Vaccinol. 2014;3:134–42. 25243025 10.1016/j.trivac.2014.06.002 PMC4165355

[18] Nelde A, Rammensee HG, Walz JS. The Peptide Vaccine of the Future. Mol Cell Proteom. 2021;20:100022. 10.1074/mcp.R120.002309 PMC7950068 33583769

[19] Kreiter S, Vormehr M, van de Roemer N, Diken M, Lower M, Diekmann J, et al. Mutant MHC class II epitopes drive therapeutic immune responses to cancer. Nature. 2015;520:692–6. 25901682 10.1038/nature14426 PMC4838069

[20] Corrales L, Gajewski TF. Molecular pathways: targeting the stimulator of interferon genes (STING) in the immunotherapy of cancer. Clin Cancer Res. 2015;21:4774–9. 26373573 10.1158/1078-0432.CCR-15-1362 PMC4750108

[21] Zhang J, Cui X, Huang Y, Xu X, Feng C, Li J. Anticancer effect of STING agonist-encapsulated liposomes on breast cancer. Molecules. 2023;28:3740. 10.3390/molecules28093740 PMC10179927 37175150

[22] Badovinac VP, Messingham KA, Jabbari A, Haring JS, Harty JT. Accelerated CD8+ T-cell memory and prime-boost response after dendritic-cell vaccination. Nat Med. 2005;11:748–56. 15951824 10.1038/nm1257

[23] de Mey W, Locy H, De Ridder K, De Schrijver P, Autaers D, Lakdimi A, et al. An mRNA mix redirects dendritic cells towards an antiviral program, inducing anticancer cytotoxic stem cell and central memory CD8(+) T cells. Front Immunol. 2023;14:1111523. 36860873 10.3389/fimmu.2023.1111523 PMC9969480

[24] Medina-Echeverz J, Ma C, Duffy AG, Eggert T, Hawk N, Kleiner DE, et al. Systemic agonistic anti-CD40 treatment of tumor-bearing mice modulates hepatic myeloid-suppressive cells and causes immune-mediated liver damage. Cancer Immunol Res. 2015;3:557–66. 25637366 10.1158/2326-6066.CIR-14-0182 PMC4420683

[25] Schmidt NW, Podyminogin RL, Butler NS, Badovinac VP, Tucker BJ, Bahjat KS, et al. Memory CD8 T cell responses exceeding a large but definable threshold provide long-term immunity to malaria. Proc Natl Acad Sci USA. 2008;105:14017–22. 18780790 10.1073/pnas.0805452105 PMC2544571

