# Supplementary figures and images for: Sequential STING and CD40 agonism drives massive expansion of tumor-specific T cells in liposomal peptide vaccines
Source: Cell Mol Immunol. 2025 Jan 1;22(2):150–60. doi: 10.1038/s41423-024-01249-4 (PMC11782543; doi:10.1038/s41423-024-01249-4)

Supplemental Figure 1

A

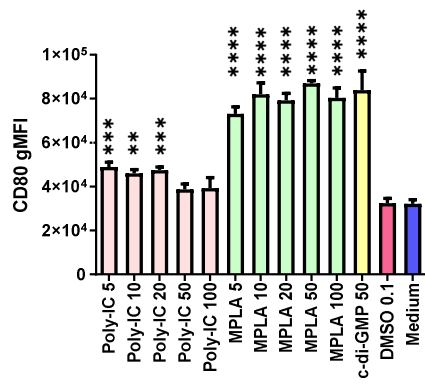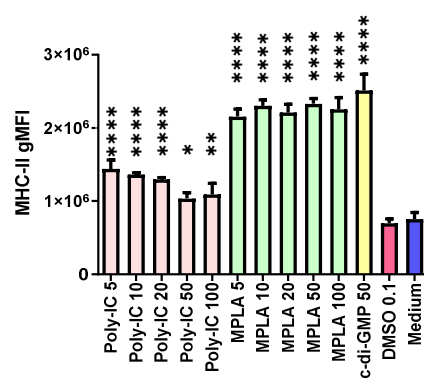

Supplement: Supplementary file 1 — Supplemental Figure 1: Different adjuvants induce the activation of cultured BMDCs [file 41423_2024_1249_MOESM1_ESM.pdf]

Supplemental Figure 2

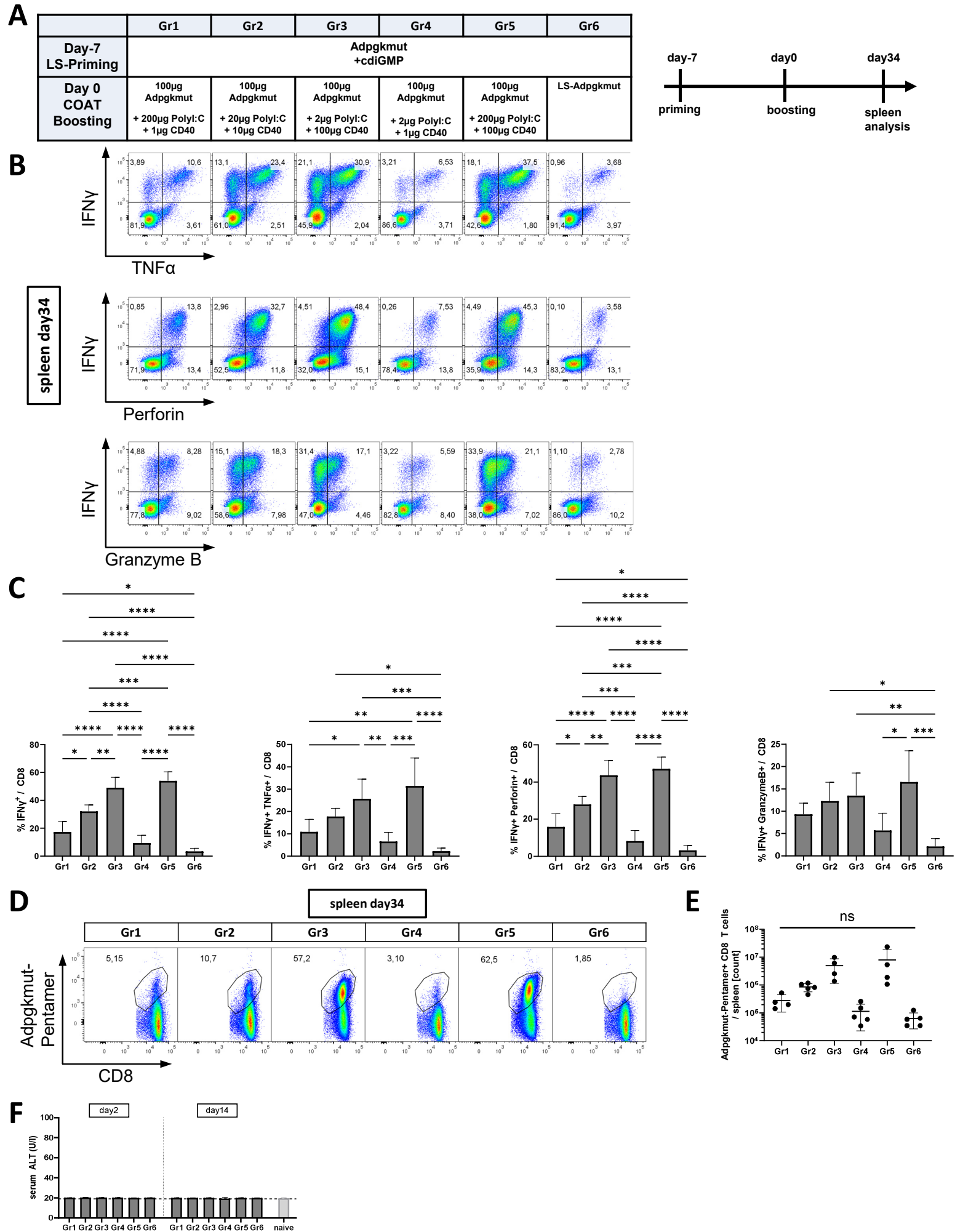

Supplement: Supplementary file 2 — Supplemental Figure 2: Tcell expansion in heterologous vaccination is mediated mainly by CD40 costimulation [file 41423_2024_1249_MOESM2_ESM.pdf]
