# Supplementary material for: Sequential STING and CD40 agonism drives massive expansion of tumor-specific T cells in liposomal peptide vaccines
Source: Cell Mol Immunol. 2025 Jan 1;22(2):150–60. doi: 10.1038/s41423-024-01249-4 (PMC11782543; doi:10.1038/s41423-024-01249-4)

Supplemental Figure 3

A

Group	Day0<br>10^7 Hep55.1C Adpgkmut s.c.	Day 7<br>Priming	Day 14<br>Boosting	ICB<br>(2x per week for 3 weeks)
LS-COAT + αPD-1	s.c. tu	Liposomes + Adpgkmut + cdiGMP +αPD-1	Adpgkmut + PolyI:C + CD40ab	αPD-1
αPD-1	s.c. tu	αPD-1	-	αPD-1
untreated	s.c. tu	-	-	-

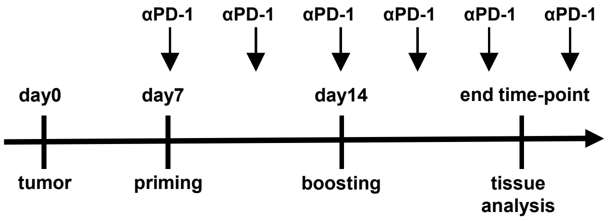

B

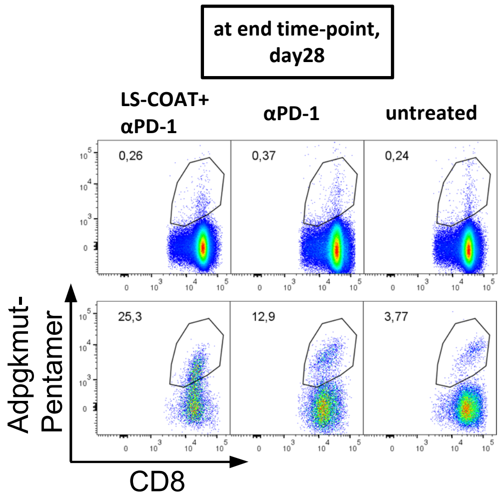

C

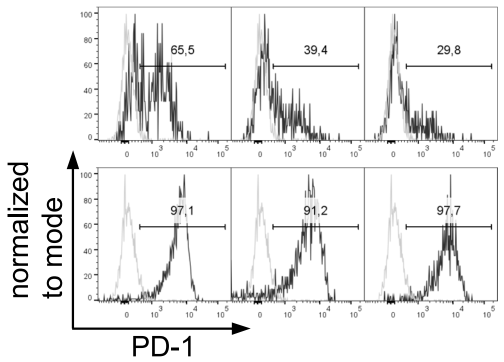

D

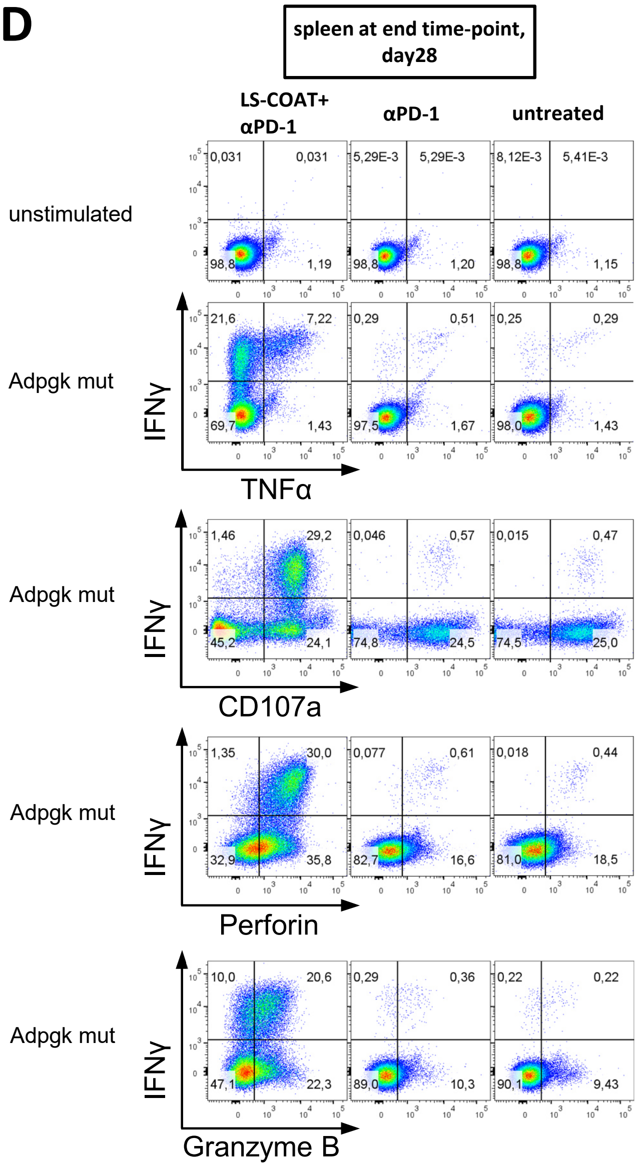

E

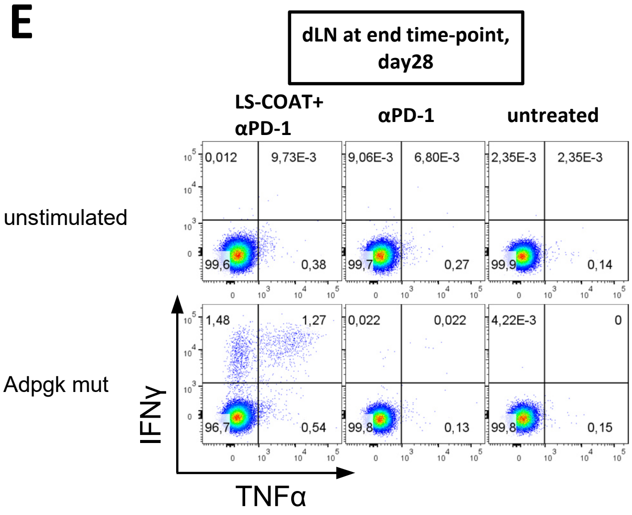

F

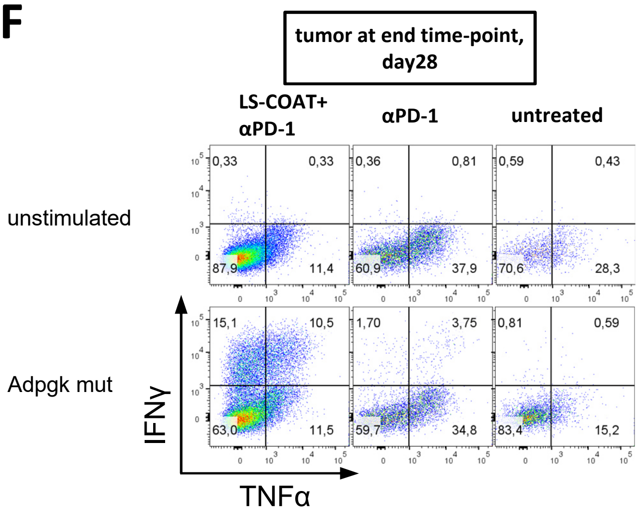

Supplement: Supplementary file 3 — Supplemental Figure 3: Anti-PD-1 monotherapy is insufficient to prevent T-cell exhaustion in the Hep-55.1C tumor model [file 41423_2024_1249_MOESM3_ESM.pdf]
