# Supplementary material for: Sequential STING and CD40 agonism drives massive expansion of tumor-specific T cells in liposomal peptide vaccines
Source: Cell Mol Immunol. 2025 Jan 1;22(2):150–60. doi: 10.1038/s41423-024-01249-4 (PMC11782543; doi:10.1038/s41423-024-01249-4)

Supplemental Figure 4

A

Group	Day0<br>10^7 Hep55.1C Adpgkmut s.c.	Day 7<br>Priming	Day 14<br>Boosting	ICB<br>(2x per week for 3 weeks)
**LS-COAT**	s.c. tu	Liposomes + Adpgkmut + cdiGMP	Adpgkmut + PolyI:C + CD40ab	-
**LS-COAT + αPD-1**	s.c. tu	Liposomes + Adpgkmut + cdiGMP **+αPD-1**	Adpgkmut + PolyI:C + CD40ab	**αPD-1**
**αPD-1**	s.c. tu	**αPD-1**	-	**αPD-1**
**untreated**	s.c. tu	-	-	-

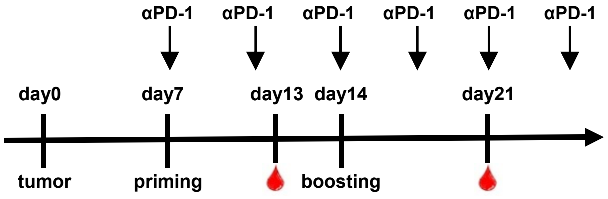

B

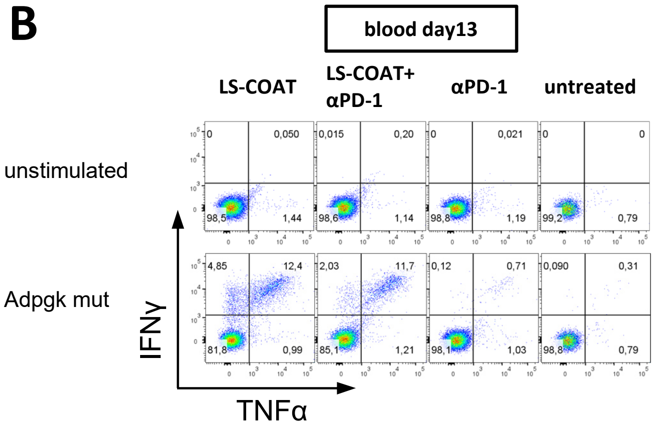

C

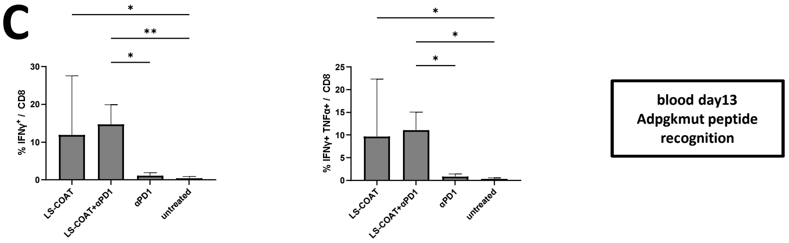

D

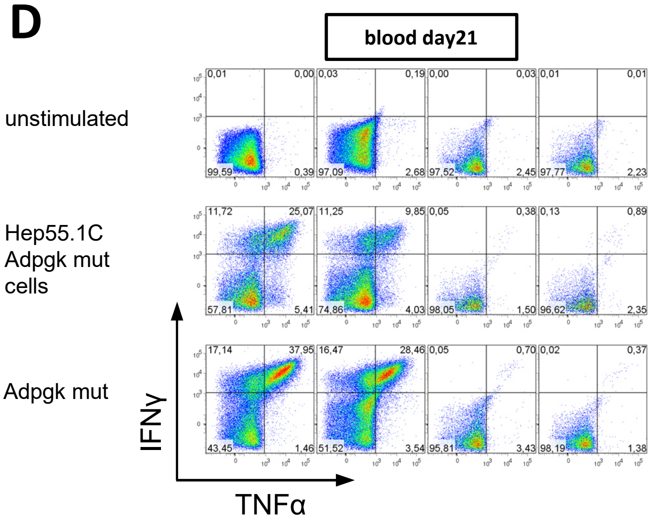

E

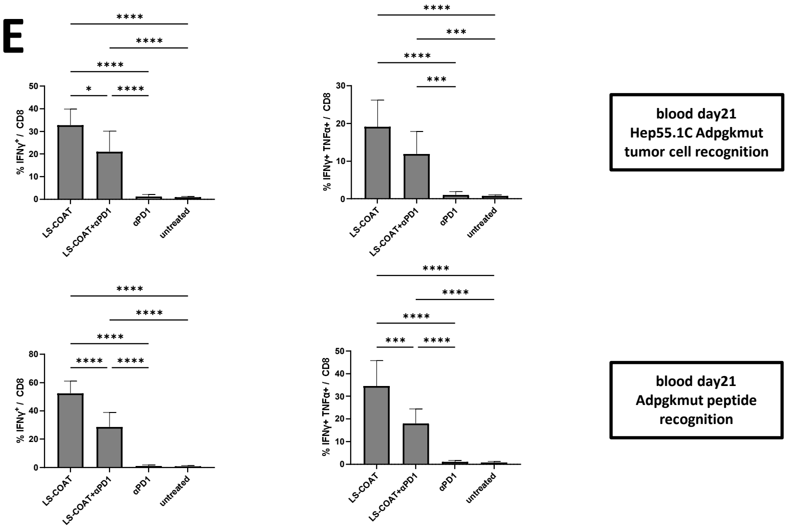

F

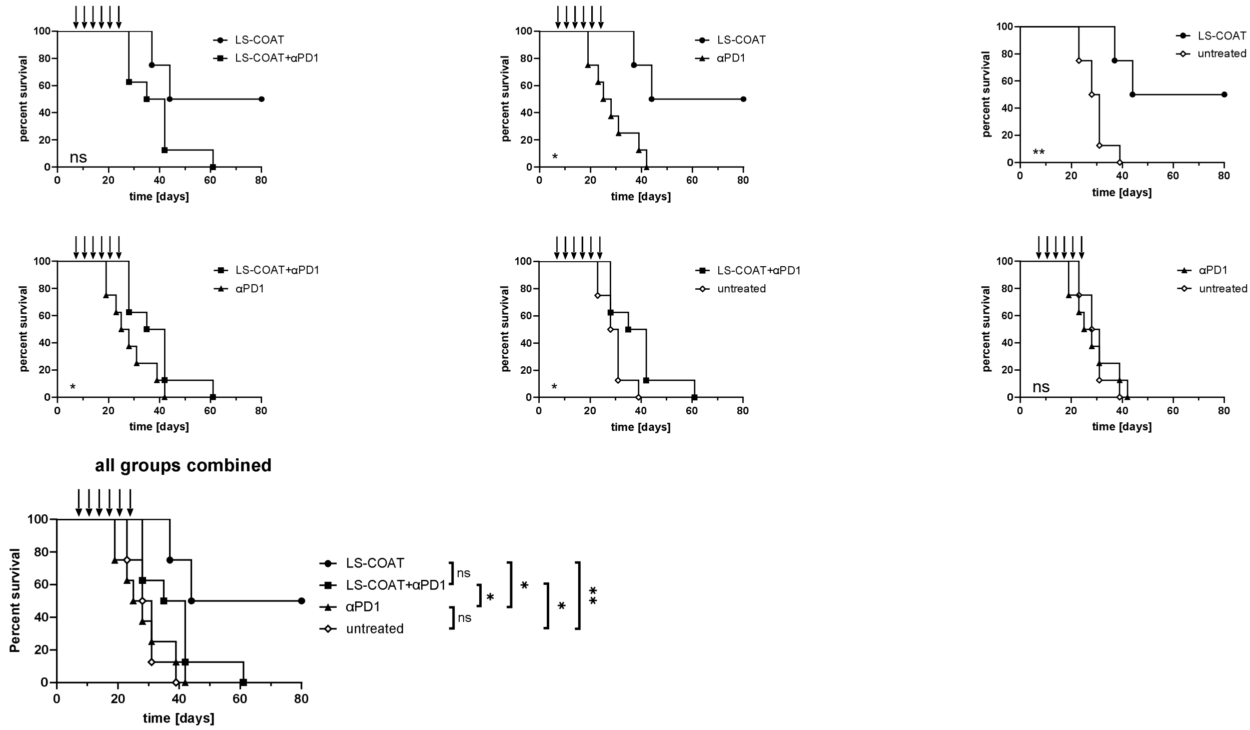

Supplement: Supplementary file 4 — Supplemental Figure 4: Therapeutic vaccination with LS-CoAT induces high T-cell responses that recognize cancer cells in vitro and prolong the survival of tumor-bearing mice [file 41423_2024_1249_MOESM4_ESM.pdf]
