# Supplementary material for: Sequential STING and CD40 agonism drives massive expansion of tumor-specific T cells in liposomal peptide vaccines
Source: Cell Mol Immunol. 2025 Jan 1;22(2):150–60. doi: 10.1038/s41423-024-01249-4 (PMC11782543; doi:10.1038/s41423-024-01249-4)

Supplemental Figure 5

A

Group	Day0 10^7 Hep55.1C Adpgkmut s.c.	Day 7 Priming	Day 14 Boosting
**LS-COAT**	s.c. tu	Liposomes + Adpgkmut + cdiGMP	Adpgkmut + PolyI:C + CD40ab
**LS-COT**	s.c. tu	Liposomes	PolyI:C + CD40ab

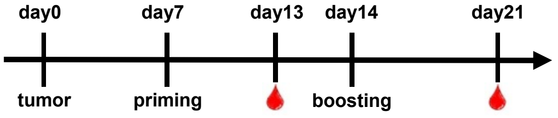

B

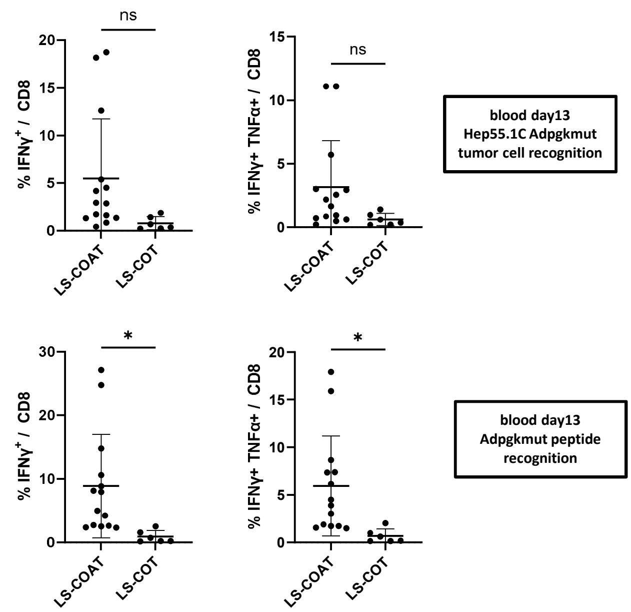

C

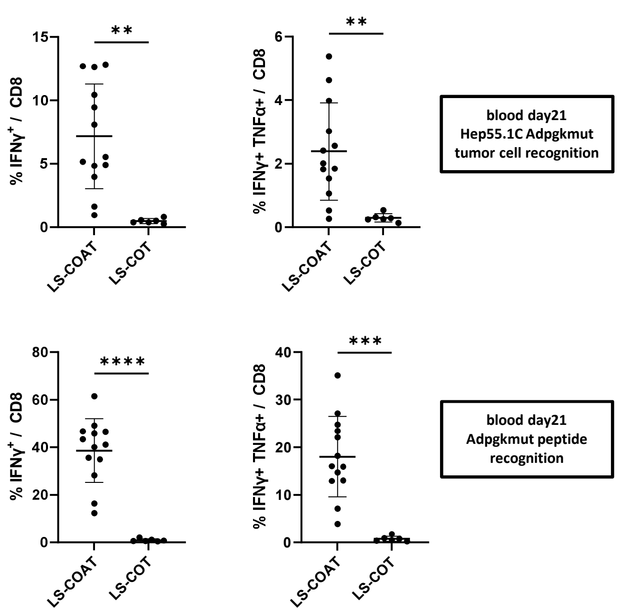

D

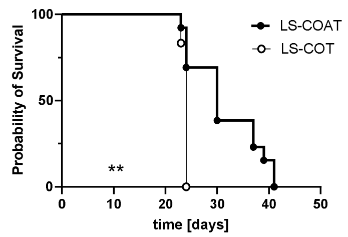

Supplement: Supplementary file 5 — Supplemental Figure 5: Tumor antigens in therapeutic T-cell vaccination are necessary for prolonged survival of tumor-bearing mice [file 41423_2024_1249_MOESM5_ESM.pdf]
