# Supplementary material for: Sequential STING and CD40 agonism drives massive expansion of tumor-specific T cells in liposomal peptide vaccines
Source: Cell Mol Immunol. 2025 Jan 1;22(2):150–60. doi: 10.1038/s41423-024-01249-4 (PMC11782543; doi:10.1038/s41423-024-01249-4)
Supplement: Supplementary file 6 — Supplemental Figure Captions [file 41423_2024_1249_MOESM6_ESM.docx]

**Supplemental Figure 1: Different adjuvants induce the activation of cultured BMDCs**

(A) Bone marrow dendritic cells were exposed to different concentrations of different adjuvants (PolyI:C (5, 10, 20, 50, or 100 µg/ml), MPLA (5, 10, 20, 50, or 100 ng/ml), or cdiGMP (50 µg/ml)) for 24 h. BMDC activation was analyzed by the upregulation of CD80 and MHCII (geometric mean fluorescence intensity (gMFI)) expression via flow cytometry. Cells treated with DMSO or medium were used as negative controls. The bars indicate the means +/- SDs of 3 samples per treatment group. Statistical analysis was performed via one-way ANOVA with Dunnett's multiple comparisons test (all compared with DMSO treated cells), p ≤ 0.05 = *, p ≤ 0.01 = **, p ≤ 0.001 = ***, p ≤ 0.0001 = ****

**Supplemental Figure 2: T-cell expansion in heterologous vaccination is mediated mainly by CD40 costimulation**

(A) C57BL/6J mice received primary immunization with liposomes containing the Adpgk^mut^ peptide and cdiGMP, followed by CoAT-boosting immunizations with different CD40 antibody and PolyI:C amounts (the amount of Adpgk^mut^ peptide remained the same), or liposomes with the Adpgk^mut^ peptide and cdiGMP, respectively. The timeline indicates measurements of samples from immunized animals. (B) On day 34, the polyfunctionality of Adpgk^mut^-specific CD8 T cells in the spleen was investigated via intracellular staining for IFNγ, TNFα, perforin and granzyme B. Dot plots show representative examples of the immune response in each group, and the numbers indicate the frequency of specific CD8 T cells in total CD8 T cells. (C) Bar graph showing the results from all the mice for cytokines (IFNγ+ or IFNγ+ and TNFα+ or IFNγ+ and perforin+ or IFNγ+ and granzyme B+) producing specific CD8+ T cells in the spleen. The bars indicate the means +/- SDs of 5 samples per group. p ≤ 0.05 = *, p ≤ 0.01 = **, p ≤ 0.001 = ***, p ≤ 0.0001 = **** (one-way ANOVA with Tukey’s multiple comparison post-hoc test). (D) Adpgk^mut^-pentamer staining was performed on day 34 using cells from the spleens of the same animals described in (A-C). Dot plots show representative examples of pentamer staining of the immune response from each group, and the numbers indicate the frequency of specific CD8+ T cells among the total CD8+ T cells. (E) Absolute numbers of Adpgk^mut^-pentamer-positive CD8 T cells were calculated on the basis of the total number of cells from the spleens of immunized mice and the frequency of Adpgk^mut^-pentamer-positive total CD8 T cells by flow cytometry on day 34 after boosting. (F) C57BL/6J mice were immunized as indicated in (A) by priming and boosting regimens. Two days and 14 days after the boost, alanine transaminase (ALT) activity was measured in the serum of immunized mice. The dotted line and gray bar indicate ALT enzyme activity in naive mice. The bars indicate the means +/- SDs of 5 samples per group.

**Supplemental Figure 3: Anti-PD-1 monotherapy is insufficient to prevent T-cell exhaustion in the Hep-55.1C tumor model**

(A) Experimental setup. C57BL/6J mice were inoculated subcutaneously with 10^7^ Hep-55.1C Adpgk^mut^-positive cells on day 0. Tumor-bearing mice received either LS-CoAT vaccination plus anti-PD-1 antibody or anti-PD-1 antibody. Untreated tumor-bearing mice were used as controls. The timeline indicates measurements of samples from immunized animals. The vertical arrows indicate αPD-1 applications. (B) Representative dot plots showing the frequency of Adpgk^mut^-pentamer-positive CD8+ T cells in draining lymph nodes and tumors at the end time-point (day 28 after tumor inoculation). (C) Histograms showing the expression of PD-1 on the surface of Adpgk^mut^-pentamer-positive CD8+ T cells in draining lymph nodes and tumors at the end time-point.The numbers indicate the percentage of PD-1-positive CD8+ T cells; gray histograms represent isotypes. (D) Representative dot plots showing cytokine expression (IFNγ+ and TNFα+ or IFNγ+ and perforin+ or IFNγ+ and granzyme B+) and degranulation capability (IFNγ+ and CD107a) of tumor-specific CD8+ T cells in the spleen at the end time-point (measured by ICS). (E-F) Representative dot plots showing cytokine expression (IFNγ+ and TNFα+) in draining lymph nodes and tumors at the end time-point.

**Supplemental Figure 4: Therapeutic vaccination with LS-CoAT induces high T-cell responses that recognize cancer cells *in vitro* and prolong the survival of tumor-bearing mice**

(A) Experimental setup. C57BL/6J mice were inoculated subcutaneously with 10^7^ Hep-55.1C Adpgk^mut^-positive cells on day 0. Tumor-bearing mice received either LS-CoAT vaccination, anti-PD-1 antibody or LS-CoAT vaccination plus anti-PD-1 antibody. Untreated tumor-bearing mice were used as controls. The timeline indicates measurements of samples from immunized animals. The vertical arrows indicate αPD-1 applications. Induced primary (day 13) and secondary (day 21) immune responses were analyzed in peripheral blood by ICS. (B) Dot plots showing representative examples of the primary (day 13) immune response in each group. The numbers indicate the frequency of specific CD8+ T cells among total CD8+ T cells (restimulated for ICS with the Adpgk^mut^ peptide). (C) Bar graph showing the results from all mice for IFNγ+ or IFNγ+ and TNFα+ producingspecific CD8+ T cells in the peripheral blood. (D) Dot plots showing representative examples of secondary (day 21) immune responses in peripheral blood from each group. The numbers indicate the frequency of specific CD8+ T cells in total CD8+ T cells (restimulated for ICS with Hep-55.1C Adpgk^mut^-expressing cells or with the Adpgk^mut^ peptide, respectively). (E) Bar graph showing the results from all mice for IFNγ+ or IFNγ+ and TNFα+ producing specific CD8+ T cells in the peripheral blood. The bars indicate the means +/- SDs of 4--8 samples per group. (F) Curvival curves of the mice from the same experiment. The arrows indicate the application of the anti-PD-1 antibody in the different groups.

**Supplemental Figure 5: Tumor antigens in therapeutic T-cell vaccination are necessary for prolonged survival of tumor-bearing mice**

(A) Experimental setup. C57BL/6J mice were inoculated subcutaneously with 10^7^ Hep-55.1C Adpgk^mut^-positive cells on day 0. Tumor-bearing mice received either LS-CoAT vaccination or LC-CoT (without antigen) vaccination. The timeline indicates measurements of samples from immunized animals. Induced primary (day 13) and secondary (day 21) immune responses were analyzed in peripheral blood by ICS. (B) Bar graph showing primary (day 13) immune responses from all mice to IFNγ+ or IFNγ+ and TNFα+ producing specific CD8+ T cells in the peripheral blood (restimulated for ICS with Hep-55.1C Adpgk^mut^-expressing cells or with the Adpgk^mut^ peptide, respectively). (C) Bar graph showing secondary (day 21) immune responses from all mice to IFNγ+ or IFNγ+ and TNFα+ producing specific CD8+ T cells in the peripheral blood (restimulated for ICS with Hep-55.1C Adpgk^mut^-expressing cells or with the Adpgk^mut^ peptide, respectively). The bars indicate the means +/- SDs of 6–14 samples per group. (D) Survival curves of mice from the same experiment.
